# PD172 Off-Label Rituximab As A First-Line Immunosuppressant Treatment For Generalized Myasthenia Gravis In Wales

**DOI:** 10.1017/S0266462324003982

**Published:** 2025-01-07

**Authors:** Clare Elliott, Gail Woodland, David Haines, James Coulson

## Abstract

**Introduction:**

Generalized myasthenia gravis (gMG) is a chronic autoimmune disorder that leads to muscle weakness and fatigue. Initial treatment is with pyridostigmine and corticosteroids, but if these are ineffective off-label immunosuppressants are used. There is some published evidence that rituximab, an anti-CD20 monoclonal antibody, may be effective in the treatment of gMG, although it is unlicensed for this condition.

**Methods:**

Evidence for rituximab as a fourth-line or later immunosuppressant treatment for refractory gMG was assessed using the One Wales Medicines (OWM) process and made available in 2019. OWM provides an access route to medicines for specific patient cohorts where no licensed treatments are routinely available. A review in 2022 identified new evidence suggesting that lower dose rituximab could be an effective first-line immunosuppressant treatment for gMG. Clinicians confirmed an unmet need, so a reassessment by OWM was considered appropriate. Clinical and cost effectiveness were assessed through a literature search, budget impact analysis, and clinical expert opinion.

**Results:**

The OWM Advisory Group assessed the evidence outlined in an evidence summary report. Clinical experts also provided the clinical context and current treatment options for patients with gMG. Overall, it was found that rituximab as a first-line treatment provided a potential improvement in patient outcomes and value for money, compared with current therapy. It was, therefore, supported for routine use in April 2023. Starting and stopping criteria for rituximab were developed in collaboration with clinical experts. All patients in Wales who meet the agreed starting criteria will now be given the option of routine treatment with rituximab.

**Conclusions:**

The OWM process allows routine access to rituximab as a first-line immunosuppressant treatment for gMG. The OWM team are collaborating with clinicians across Wales to instigate a new systematic method for collecting and analyzing patient outcomes. These real-world data will be used to assess benefit to ensure continued access to the best treatments for Welsh patients.

